# Evaluation of phosphorus sources in tomato plants inoculated with plant growth-promoting rhizobacteria

**DOI:** 10.7717/peerj.20651

**Published:** 2026-02-16

**Authors:** Marco Polo Carballo-Sánchez, Juan Jose Almaraz-Suarez, Sara Monzerrat Ramírez-Olvera

**Affiliations:** Edaphology, Colegio de Postgraduados, Texcoco, State of Mexico, Mexico

**Keywords:** Hydroponics, Phosphate rock, Rhizobacteria, Tomato

## Abstract

Tomatoes are agriculturally and gastronomically significant and serve as model organisms in scientific research. This study examined plant-phosphorus interactions, evaluated two P sources for fertilization, and analyzed the effects of rhizobacteria on plant growth. Phosphorus (P) is an essential yet limited nutrient for plants. Microbial inoculants formulated with plant growth-promoting rhizobacteria (PGPR) enhance plant health and growth and improve P solubility. Tomato seedlings were inoculated with the following PGPR strains capable of P solubilization: *Paenibacillus spp*. BSP 1.1, *Arthrobacter enclensis* JN24, and *Arthrobacter pokkalii* JLB4. Morphological and physiological analyses were used to assess nitrogen (N) and P intake and developmental differences among treatments. The P source, bacterial strain, and their interactions influenced plant development differently. Plants treated with phosphate rock exhibited a higher greenness index and root volume, whereas those with soluble phosphate had increased leaf area when inoculated with BSP and JLB4. Strain JLB4 specifically increased leaf area when combined with soluble phosphate. P concentration was lower in phosphate rock-treated plants, although deficiency symptoms were absent. N concentrations and growth-related variables were diminished in the early stages but improved to the end of the experiment. In conclusion, P demand was constant, but its availability increased with the effect of P solubilization throughout the experiment. Therefore, the addition of phosphate rock as a source of P in soilless agriculture may be a viable alternative for tomato cultivation.

## Introduction

Currently, new sources of nutrients for crops are being sought because of factors that directly affect agriculture and threaten food security, including population growth, climate change, and fertilizer costs (which have increased significantly in recent years), as well as environmental problems such as soil degradation, loss of fertility, and excess fertilizer leaching to water bodies, leading to algal blooms and eutrophication (Wu & Ge, 2019). Another factor is the depletion of traditional fertilizer sources, as elements such as nitrogen (N), potassium (K), and phosphorus (P) originate from non-renewable sources, such as phosphate rock, which contains phosphorus in unavailable forms for plants, such as apatites, which require further processes to reach availability.

Among the essential nutrients, P is particularly important because of its role in cellular and metabolic processes. It plays a key role in nucleic acid structure, several metabolic functions performed by adenosine phosphates, cell membrane phospholipid function, and phytate production (Bechtaoui et al., 2021). This element is characterized by its low mobility in the soil because it is commonly found in forms that cannot be assimilated by plants, mainly due to its solubility. To address the issue of P unavailability, the current trend is to use microbial inoculants formulated with plant growth-promoting rhizobacteria (PGPR) (Tian et al., 2021). These bacteria can not only interact with plants to promote health and growth but also enhance their solubility by producing organic acids. In this study, we chose tomato plants (*Solanum licopersicum*), which are remarkably relevant to agriculture and the food industry because of their high yield, versatility in production, and demand in domestic and international markets. In addition to its high nutrient content and as a staple food in many cuisines worldwide, it is also considered a model organism for genetic and biotechnological studies, particularly in the case of interactions between plants and beneficial organisms (Quinet et al., 2019). This study examined P interactions with plants related to uptake in leaves during experimental time, evaluated two different P sources for fertilization (a common soluble fertilizer and insoluble phosphate rock), and analyzed bacterial effects on plant growth, mainly how phosphorus rock interacts with the plants, inoculated or not, compared to treatments with soluble phosphate in a nutrient solution.

## Materials and Methods

### Experiment establishment

The experiments were carried out between June and August 2024 in a greenhouse at the Colegio de Postgraduados facilities (latitude, 19°30.829′N; longitude, 98°52.729′W; altitude, 2,251 m), with an average temperature of 28.5 °C and a relative humidity of 46.5%. The substrate used was tezontle, a reddish inert stone material with a non-significant nutritional supplement to the cultures, derived from volcanic activity that is easily obtained in central Mexico and is widely used in hydroponics. Thirty-day-old tomato (*S. lycopersicum*) cv. Rio Grande seedlings from commercial seeds (Caloro, Mexico), adapted to weather and altitude in central Mexico, were transplanted into polyethylene agricultural black bags with a 2-liter capacity under two different conditions: two kg of tezontle processed with a 10-mesh sieve as a substrate for plants; bags were filled by weight using a commercial scale for reference. Seedlings were transplanted one month after sowing and watered with Steiner’s (1984) universal nutrient solution, with a composition of 12.0, 1.0, 7.0, 7.0, 9.0 and 4.0 meq⋅ L^−^^1^ of nitrate (NO_3_^−^), phosphate (H_2_PO_4_^−^), sulfate (SO_4_^2^^−^), potassium (K^+^), calcium (Ca^2^^+^) and magnesium (Mg^2^^+^), respectively, as well a commercial micronutrient mix containing B and Mo, and chelated Fe, Mn, Zn, and Cu (Ultrasol^®^ micro BSP mix; SQM nutrition, Santiago, Chile) at concentrations of 25 and 75%. The nutrient concentration in the formula varied from 40 to 70%, according to the phenological stage. This formula contains a regular source of P. Then, two kg of the same tezontle was mixed with ground phosphate rock and processed on a 100-mesh sieve at 10% (w/w) for treatment with a Steiner nutrient solution that did not contain KH_2_PO_4_, with a clear intention to use phosphate rock as the only P source. The nutrient composition of the ground phosphate rock, expressed as a percentage, was *N* = 0.06%, *P* = 10.90%, and *K* = 0.13%.

### Experimental design

The experiment consisted of a 2^4^ factorial design, in which the effects of two P sources, three bacterial strains, and a control without bacterial inoculation were determined.

### Inoculation of bacterial strains

Seven days after transplantation (DAT), the seedlings were inoculated with the following strains from the Soil Microbiology Laboratory collection, whose P solubilization capacity has already been reported, and were assigned to the species level by sequencing 16s  rDNA, which have been deposited in GenBank with their respective accession numbers: *Paenibacillus terrae* BSP 1.1 (PX724054), *Arthrobacter enclensis* JN24 (MW629812), and *Arthrobacter pokkalii* JLB4 (MW629814). Inoculation was performed on the roots of the plants with 10 ml of nutrient broth with suspended cells after 48 h of incubation at 28 °C, with a concentration of more than 10^8^ cells per ml of solution, counted in a Neubauer chamber.

### Determination of morphological and physiological parameters

The time-course parameters were determined 7, 14, 21, and 28 days after inoculation (DAI), as described below. Stem diameter was determined using a HER-411 digital caliper (Steren, Mexico) expressed in millimeters (mm). Plant height was determined using a graduated ruler expressed in centimeters (cm), and the number of leaves was counted manually. The greenness index was determined using a SPAD 502 colorimeter (Minolta, Japan) and expressed in arbitrary Soil Plant Analysis Development (SPAD) units.

Plants were harvested 28 days after inoculation (DAI), and the treatments and determinations were performed on the different organs at the end of the plant cycle (endpoint parameters).

For the aerial parts, fresh weight was determined using a portable scale and expressed in grams. Leaves were separated from the stems of each plant, and the leaf area was determined exclusively from the leaves using a Li-Cor 3100C leaf area analyzer (Li-Cor, Lincoln, NE, USA) and expressed in square centimeters (cm^2^). Photopigment concentrations were determined spectrophotometrically at 470, 645, and 660 nm for chlorophyll a, chlorophyll b, and carotenoids, respectively. Subsequently, the leaves and stems were bagged separately and dried in a forced-air oven at 70 °C for two days. The dry weights of the leaves and stems were determined using a laboratory balance and expressed in grams (g). The leaves were then ground in a spice mill and sieved for N, P, and K analysis.

Nutrient analysis was performed at the Environmental Analysis Laboratory of the same institution where the authors are affiliated, with the methods described by Benton, Wolf & Mills (1991) and Reuter et al. (1986). For N analysis, digestion was performed using H_2_SO_4_, and determination was performed using the Kjeldahl method *via* steam distillation and titration. For P and K analysis, HNO_3_ + HClO_4_ 2:1 digestion was performed, using the molybdate-vanadate spectrophotometric method for the former and flame emission spectroscopy for the latter.

The roots were separated from the aerial parts and washed to remove excess substrate. The fresh weight was determined using a portable balance. Root volume was determined using a graduated cylinder according to the volume displaced in milliliters (mL). Subsequently, the roots were dried in a forced-air oven at 70 °C for two days. The dry weight of the roots was determined using an analytical balance and expressed in grams (g).

### Statistical analysis

Analysis related to the data of the factorial experiment was performed using SAS 9.0 software (SAS Institute, Cary, NC, USA), considering a factorial ANOVA analysis, followed by Tukey’s *post-hoc* test (*p* < 0.05) for data that complied with ANOVA assumptions.

## Results

### P source factor

The P source factor significantly influenced plant height, stem diameter, number of leaves, greenness index, chlorophyll a and b, carotenoids, leaf area, biomass, root length and volume, and N and P concentrations (Table 1).

**Figure 1:**
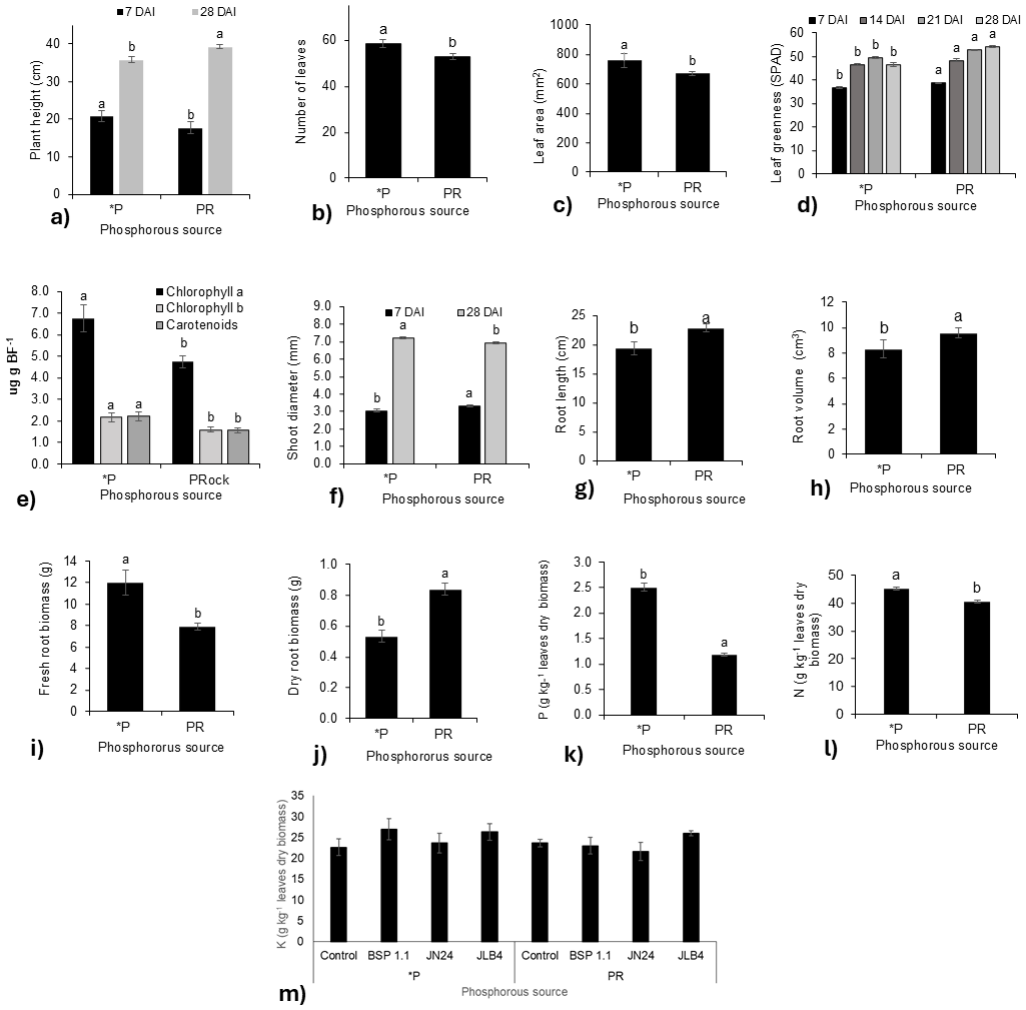
Phosphorous source factors of the variables with significant differences and potassium content in dry leaves. (A) Plant height at 7 and 28 DAI, (B) number of leaves at 21 DAI, (C) leaf area, (D) leaf greenness at 7, 14, 21, and 28 DAI, (E) Concentration of Chlorophyll a, Chlorophyll b, and carotenoids in fresh leaf biomass; (F) shoot diameter at 7 and 28 DAI; (G) root length; (H) root volume; (I) fresh root biomass; (J) dry root biomass; (K) phosphorous content in dry leaves; (L) nitrogen content in dry leaves m) potassium content in dry leaves. Legends: DAI, Days after inoculation; *P = soluble phosphate (${\mathrm{PO}}_{4}^{3+}$), PRock, Phosphorus rock, n/s = no strain inoculated. Means ± standard error with different letters in each column indicate statistical differences among the treatments (Tukey, *P* ≤ 0.05).

Plant height was significantly influenced by the phosphorus source at 7 and 28 DAI. At 7 DAI, plants fertilized with the phosphate solution were taller than those treated with phosphate rock, whereas at 28 DAI, this effect was reversed, and the height of plants treated with phosphate rock was greater (Fig. 1A). Similarly, the number of leaves (Fig. 1B) and leaf area (Fig. 1C) at 21 and 28 DAI were higher in plants fertilized with the phosphate solution.

**Table 1:** Significance of variables compared to phosphorus source (P), bacterial strain (S), and phosphorus source * bacterial strain (P+S). Variables were determined at 7, 14, 21, and 28 days after inoculation (DAI), and those that were determined at 28 DAI.

DAI	PH	SD	NL	SPAD	Chl *a*	Chl *b*	CA	LA	APFW	APDB	RFB	RDB	RV	RL	NAP	PhAP	KAP
Phosphorus source (P)																	
7	^*^	^*^	ns	^*^	–	–	–	–	–	–	–	–	–	–	–	–	–
14	ns	ns	ns	^*^	–	–	–	–	–	–	–	–	–	–	–	–	–
21	ns	ns	^*^	^*^	–	–	–	–	–	–	–	–	–	–	–	–	–
28	^*^	^*^	–	^*^	^*^	^*^	^*^	^*^	ns	ns	^*^	^*^	^*^	^*^	^*^	^*^	ns
Bacterial strain (S)																	
7	ns	ns	ns	ns	–	–	–	–	–	–	–	–	–	–	–	–	–
14	ns	^*^	^*^	ns	–	–	–	–	–	–	–	–	–	–	–	–	–
21	ns	ns	^*^	^*^	–	–	–	–	–	–	–	–	–	–	–	–	–
28	^*^	ns	–	^*^	ns	ns	^*^	^*^	^*^	^*^	ns	^*^	^*^	^*^	ns	ns	ns
Interaction (P×S)																	
7	^*^	ns	^*^	ns	–	–	–	–	–	–	–	–	–	–	–	–	–
14	ns	ns	ns	ns	–	–	–	–	–	–	–	–	–	–	–	–	–
21	ns	ns	^*^	^*^	–	–	–	–	–	–	–	–	–	–	–	–	–
28	^*^	ns	–	ns	ns	ns	ns	^*^	^*^	ns	ns	ns	^*^	^*^	^*^	^*^	ns

**Notes.**

Significant differences *p* ≤ 0.05.

* non-significant differences (ns), no data registered (-).

DAI Days after inoculation PH plant height SD shoot diameter NL number of leaves SPAD chlorophyll content index Chl a chlorophyll a Chl b chlorophyll b CA carotenoids LA leaf area APFW aerial part fresh weight APDB aerial part dry biomass RFB root fresh biomass RDB root dry biomass RV root volume RL root length NPA nitrogen in aerial part PhAP phosphorus in aerial part KAP potassium in aerial part

The leaf greenness index (Fig. 1D) was significantly higher in plants fertilized with phosphate rock at 7, 14, 21, and 28 DAI than in those treated with phosphate solution. In contrast, the concentrations of chlorophyll a, b, and carotenoids were lower than those in plants fertilized with the phosphate solution (Fig. 1E). Similarly, stem diameter was greater in plants treated with phosphate rock at 7 DAI, although at 28 DAI, the plants fertilized with the phosphate solution presented a greater diameter (Fig. 1F).

Regarding the root system, plants treated with phosphate rock developed longer roots with greater volumes (Figs. 1G, 1H). However, the phosphate solution favored greater production of root biomass, both fresh (Fig. 1I) and dry (Fig. 1J). In addition, the N (Fig. 1K) and P (Fig. 1L) concentrations in plant tissues were significantly higher in plants fertilized with the phosphate solution than in those fertilized with phosphate rock.

### Bacterial strain factor

Regarding the effect of the bacterial strain factor, significance was obtained in the variables plant height, stem diameter, number of leaves, leaf greenness index, carotenoids, leaf area, fresh and dry weight of the aerial part, root dry weight, root volume, and root length (Table 1).

**Figure 2:**
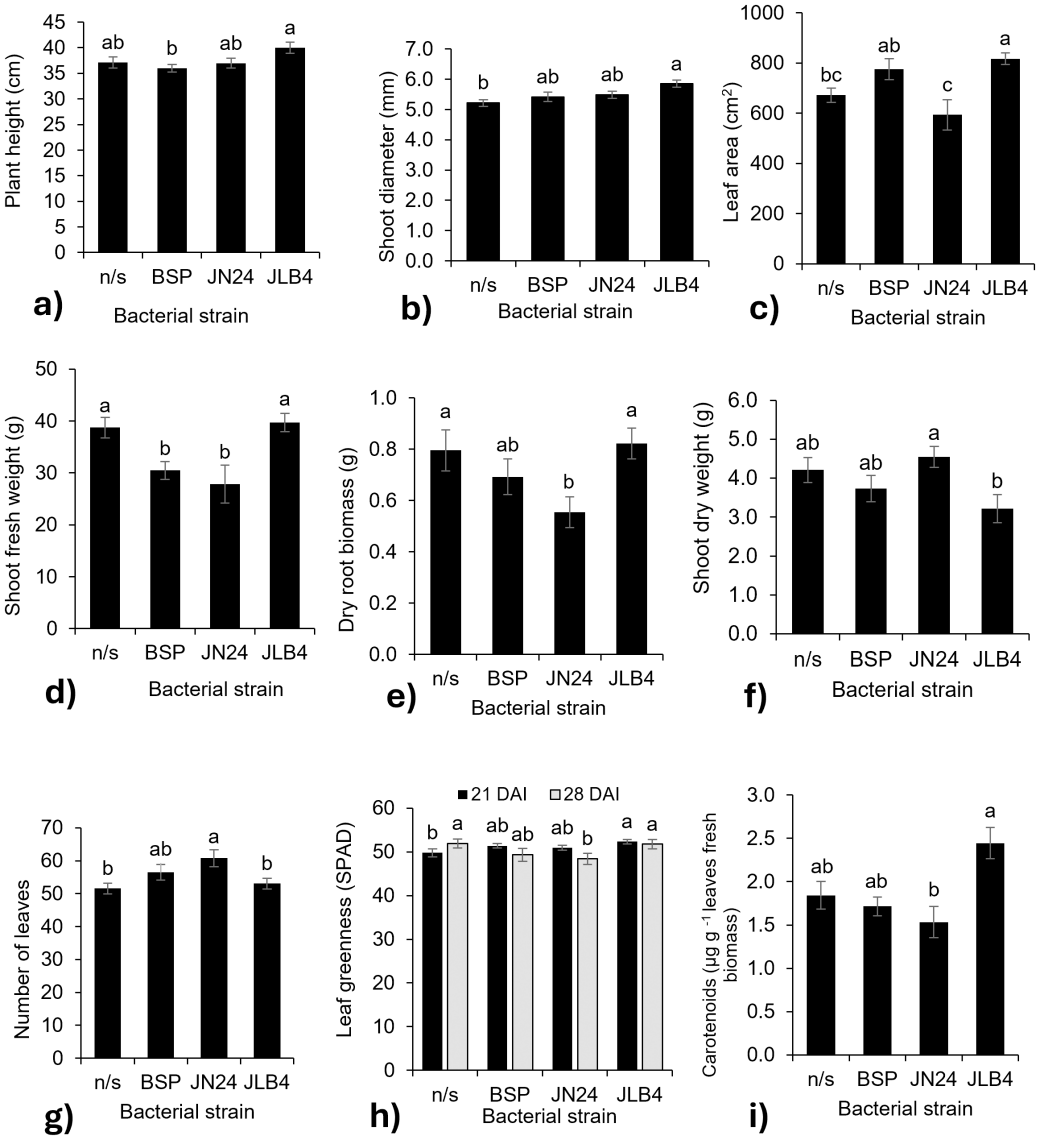
Bacterial strain factors of the variables with significant differences. (A) Plant height at 28 DAI, (B) shoot diameter at 14 DAI, (C) leaf area, (D) shoot fresh weight, (E) dry root biomass, (F) shoot dry weight, (G) number of leaves at 21 DAI, (H) leaf greenness at 21 and 28 DAI, and (I) concentration of carotenoids in fresh leaves, and legends: DAI, Days after inoculation, *P, soluble phosphate (${\mathrm{PO}}_{4}^{3+}$), PRock, Phosphorus rock, n/s, no strain inoculated. Means ± standard error with different letters in each column indicate statistical differences among the treatments (Tukey, *P* ≤ 0.05).

The height of plants inoculated with JLB4 at 28 DAI was greater than that of plants inoculated with BSP; however, no strain was significantly different from that of the non-inoculated plants (Fig. 2A). Stem diameter (Fig. 2B) and leaf area (Fig. 2C) were significantly greater in plants inoculated with JLB4 than in non-inoculated plants.

In contrast, the fresh weight of the aerial parts decreased with the inoculation of strains BSP and JN24 (Fig. 2D), as well as the root dry biomass weight (Fig. 2E) with the inoculation of strain JN24. The dry weight of the aerial parts was not significantly altered after inoculation with the bacterial strains (Fig. 2F), but this variable was significantly higher after inoculation with JN24 than with JLB4. In contrast, the number of leaves was significantly higher in plants inoculated with strain JN24 (Fig. 2G) than in non-inoculated plants. Regarding the leaf greenness index, JLB4 significantly increased this variable at 21 DAI compared to that in non-inoculated plants. Inoculation with JN24 decreased this variable at 28 DAI compared to that in non-inoculated plants (Fig. 2H). Similarly, carotenoid concentrations were higher in plants inoculated with JLB4 than in those inoculated with JN24, with no significant effects observed in non-inoculated plants (Fig. 2I).

### P source and bacterial strain interaction

The interaction between P source and bacterial strain was significant for plant height, number of leaves, greenness index, leaf area, fresh weight of the aerial part, root length and volume, and N and P concentrations (Table 1).

**Figure 3:**
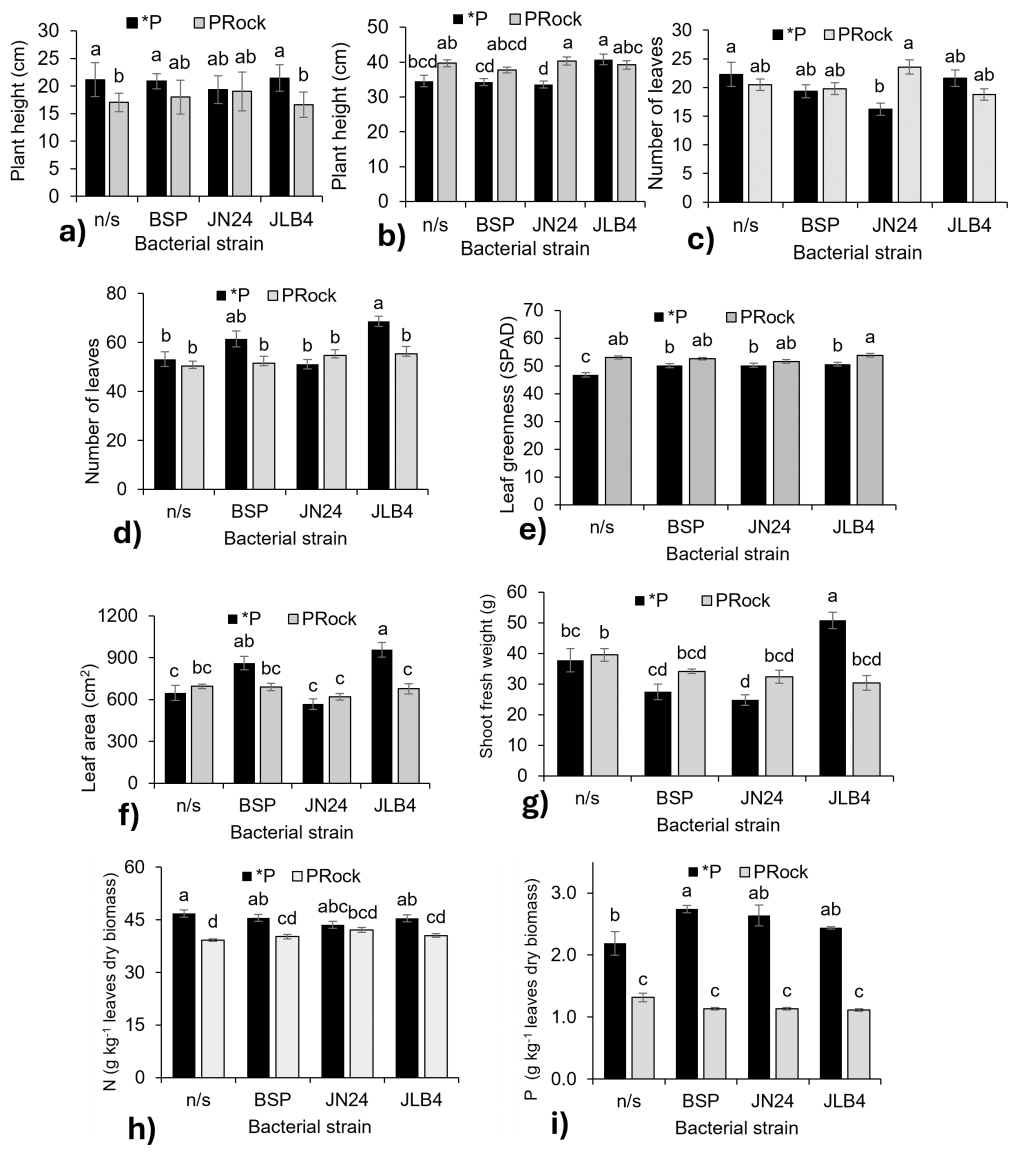
Strain × phosphorus source factor interaction of variables with significant differences. (A) Plant height at 7 DAI, (B) plant height at 28 DAI, (C) number of leaves at 7 DAI, (D) number of leaves at 21 DAI, (E) leaf greenness at 21 DAI, (F) leaf area, (G) shoot fresh weight, (H) nitrogen content in dry leaves, and (I) phosphorous content in dry leaves. *P, soluble phosphate (${\mathrm{PO}}_{4}^{3+}$), PRock, Phosphorus rock, n/s, no strain inoculated. Means ± standard error with different letters in each column indicate statistical differences among the treatments (Tukey, *P* ≤ 0.05).

At 7 DAI (Fig. 3A), non-inoculated plants fertilized with phosphate rock were shorter than those receiving phosphate. Plants inoculated with BSP and JN24 showed no significant differences between the phosphorus sources. In contrast, the combination of JLB4 and the phosphate rock significantly decreased this parameter. At 28 DAI (Fig. 3B), non-inoculated plants showed no significant changes in height. However, those treated with JN24 and phosphate rock were taller than those treated with soluble phosphate alone.

Inoculation with JN24 in combination with soluble phosphate reduced the number of leaves compared to non-inoculated plants. In contrast, no significant differences were observed when combined with phosphate rock (Fig. 3C). At 21 DAI, the application of soluble phosphate together with strain JLB4 significantly increased the number of leaves compared to non-inoculated plants, whereas with phosphate rock as a P source, no significant effects on this variable were detected (Fig. 3D).

Bacterial strains BSP, JN24, and JLB4 significantly increased the leaf greenness index when plants were supplied with soluble phosphate, without significantly altering this variable when phosphate rock was used as the P source (Fig. 3E).

Leaf area was significantly higher in plants inoculated with BSP and JLB4 in combination with soluble phosphate than in non-inoculated plants. Strain BSP showed no differences in this variable between the two phosphorus sources, whereas strain JLB4 increased leaf area when applied with soluble phosphate (Fig. 3F). Similarly, shoot fresh weight increased significantly after inoculation with strain JLB4 and phosphate solution supply compared with non-inoculated plants that also received phosphate. However, this effect was not observed when JLB4 was combined with phosphate rock (Fig. 3G).

### N, P, and K concentration

The N concentration was lower in non-inoculated plants that received phosphate rock. Similarly, plants inoculated with BSP and JN24 in combination with phosphate rock had lower N concentrations than those treated with phosphate. However, in plants inoculated with JN24, N concentration did not vary between the two P sources (Fig. 3H).

Plants that were not inoculated and fertilized with phosphate had higher P concentrations than those that were not inoculated and treated with phosphate rock. In addition, inoculation with the BSP strain in combination with soluble phosphate significantly increased P concentration compared to non-inoculated plants. In contrast, JN24 and JLB4 did not modify this variable under any phosphorus source (Fig. 3I).

In addition, no significant differences were found in the K concentration in the tissue for all treatments (Fig. 1K).

## Discussion

### P availability

The results of this study highlight the importance of P sources and their interactions with bacterial strains in tomato growth and development. The use of soluble phosphate promoted plant growth, as reflected by greater height, leaf number, leaf area, biomass, pigment, and nutrient concentration. This is because of the high solubility of phosphate fertilizers, which allows immediate availability of phosphorus to the plant (Chien et al., 2011; Poirier, Jaskolowski & Clúa, 2022). In the early stages, plants treated with soluble P outperformed in growth compared to those treated with phosphate rock. This is because of the high demand for P in young plants compared to mature plants (Malhotra, Vandana Sharma & Pandey, 2018), which is due to the structural role of P as a component of biomolecules and its participation in key processes of plant metabolism (Lambers, 2022).

The main limitation of the direct use of phosphate rock is its low availability for plant assimilation, particularly in soils with neutral or alkaline pH values. To improve its effectiveness, strategies have been developed to increase its solubility, such as acidification of the environment, which favors P release (Fayiga & Nwoke, 2016). According to Pita-Barbosa et al. (2025), during an experiment conducted on tomatoes, root exudation was altered in response to P deficiency in terms of quantity and composition. The metabolites that are outstanding during this stress stage are proteins, phenolic compounds, and carboxylates, mainly lactate, malate, fumarate, and oxalate. These organic acids contribute to inorganic P solubilization, which may explain the non-significant differences in P concentration in leaves in phosphate rock treatments, in both inoculated and non-strain plants (Fig. 3I). However, the use of phosphate rock, known for its low solubility (Fayiga & Nwoke, 2016), showed differentiated behavior. Although positive effects were observed in some variables, such as the greenness index and stem diameter at 7 DAI, where there was a notable increase in treatments with phosphate rock. The greenness index in plants is relevant to plant physiology as an estimate of chlorophyll content in plant leaves. The main effect of a physiological response to low P availability is enhanced chlorophyll accumulation in leaves (Jaisue et al., 2025) because of the essential role of P in the photosynthetic process (Bechtaoui et al., 2021; Nam et al., 2021). However, no increase in pigment concentration was observed in the phosphate rock treatments compared to the soluble phosphate treatments (Fig. 1E). The reasons for the increase in greenness index values, despite the lack of chlorophyll accumulation or increased concentration, are as follows: changes in leaf intracellular water tend to decrease mesophyll cell volume (Zhang et al., 2024), structural disorganization of chloroplasts (Henningsen et al., 2023), leaf thinning affects solar spectral reflectance and transmittance of natural leaves (Xu & Ye, 2023), as well as internal scattering (Xiong et al., 2015).

### Bacterial inoculation

Biological approaches have also gained attention for enhancing P availability in plants. Phosphate-solubilizing microorganisms play a crucial role in transforming insoluble P into plant-accessible forms (Rawat et al., 2021). In addition, these microorganisms produce hormones that stimulate plant growth by intervening in processes such as cell division, root development, flowering, and germination (Puri, Padda & Chanway, 2020). Similarly, the microbiota can mineralize and gradually release P (Timofeeva, Galyamova & Sedykh, 2022).

Inoculation with bacterial strains increases tomato growth. However, the results varied depending on the strain. Strain JLB4 showed a clear and consistent promoter effect, increasing plant height, leaf area, root volume, and root length, and significantly increasing biomass. This behavior suggests the involvement of mechanisms such as P solubilization and phytohormone production, which stimulate cell division and elongation. In contrast to JLB4 and JN24 showed a limited effect, with partial improvements in leaf number and development. In contrast, BSP 1.1 strain did not provide significant benefits, and in some cases, reduced certain growth variables. This indicates that the effectiveness of microorganisms depends on their specific capabilities and interactions with the crop environment.

The interaction between the P source and bacterial inoculation was a key factor in the tomato response. In the short term, the combination of phosphate rock with some strains, such as JN24, showed a compensatory effect by facilitating P solubilization. In contrast, the joint application of soluble phosphate and JLB4 resulted in synergistic effects, as reflected in a higher biomass dry weight. This suggests that a highly available P source is essential for maximizing the biostimulatory potential of bacteria.

### N, P and K concentrations in tissue

In this study, comparisons between treatments with different P sources led to a situation in which the P concentration in the tissue was lower in the phosphorus rock treatments. The P concentration in the tissue was significantly higher in plants fertilized with phosphate than in those receiving phosphate rock. Phosphate in soluble form, as in fertilizers, facilitates its uptake and accumulation in plants. This situation, which may lead us to think about a disadvantage when using phosphorus rock, is not a problem when the PGPR facilitates nutrient uptake. First, there was no evidence of P deficiency in the aerial parts of these plants, such as the purple leaves. Second, there were no proteoid roots in the phosphate rock treatment. Third, during P deficiency, root growth and length are privileged and aerial part growth is limited. In this experiment, the plant height variable in phosphate rock treatments was lower at 7 DAI, but at 28 DAI was higher than that in soluble potassium phosphate treatments, and the treatment that resulted in the highest height was the JN24 strain (Figs. 3A, 3B). Regarding root volume and length, higher results were obtained in the phosphate rock treatments, which can be observed in the increase in root length and dry root biomass (Figs. 1G, 1J). During P deficiency, it tends to be translocated to the root to increase survival possibilities, as well the increase of longer root hairs and lateral roots production to increase P uptake (Khan et al., 2023). However, the combination with JLB4 significantly increased the dry root biomass, chlorophyll, and carotenoid concentrations, as well as N and P accumulation in plants. Notably, increases in root volume and root dry weight have been reported previously in different studies by the effect of the JLB4 strain (Daza-Martínez et al., 2022; Miguel-Cruz et al., 2024).

For K concentration, no significant differences were observed, and there was no deficiency observed, since the supply was the same for all treatments, and no disruption in uptake may have occurred in plants.

Referring to the N concentration in tissue, the supply of phosphate rock resulted in a slightly lower concentration of this nutrient than in the potassium phosphate treatments, considering that the N supply was the same for both, but with no signs of N deficiency and no significant effects related to this situation. The P deficiency affects the uptake of N (Khan et al., 2023), this may be explained by the interdependency of both nutrients in plant metabolism. Cytokinin levels decrease and induce an accumulation of N in the roots, which leads to negative feedback that inhibits N uptake. P limitation causes export of sugars to the root, reducing the pool of carbohydrates for N assimilation and increasing asparagine levels in roots and stems (Mishra et al., 2024). To alleviate N deficiency, PGPR may contribute not only to P solubilization but also to cytokinin production. Martynenko et al. (2022) reported that a cytokinin producer *Bacillus subtilis* strain prevented the decline in both potassium and phosphorus concentrations and increased concentration of cytokinins in salt-stressed plants. Khoso et al. (2024) reports that bacteria from genus *Arthrobacter, Azospirillum, Bradyrhizobium, Bacillus, Pseudomonas*, and *Paenibacillus* can synthesize cytokinins for plant stress alleviation. This synthesis capacity is important for root colonization, and PGPR-related *ipt* genes are clustered with genes of other enzymes related to the CK biosynthetic pathway (Timofeeva, Galyamova & Sedykh, 2024).

## Conclusions

The effects of rhizobacteria on tomato plant development were observed under different phosphorus sources and different bacterial strains were inoculated. Although the effect of phosphate rock was not evident in the first weeks of the vegetative stage after inoculation when compared with that of the soluble P source, it increased in the last weeks of the experiment. P demand was constant, but its availability increased with the effect of P solubilization induced by inoculation throughout the experiment. Therefore, the effect of PGPR inoculation and the addition of phosphate rock as a source of P in soilless agriculture may be a viable alternative for tomato cultivation.

##  Supplemental Information

10.7717/peerj.20651/supp-1 Supplemental Information 1 Significance analysis of factors P hosphorous source, bacterial strain, and the interaction of both factors related to plant height, shoot diameter, and number of leaves in tomato (*Solanum lycopersicum*).

10.7717/peerj.20651/supp-2 Supplemental Information 2 Raw data

## Additional Information and Declarations

### Data Availability

The following information was supplied regarding data availability: The raw data is available in the Supplemental File. The raw sequence data are available at NCBI GenBank: MW629812, MW629814, PX724054.
