# Supplementary material for: Evaluation of phosphorus sources in tomato plants inoculated with plant growth-promoting rhizobacteria
Source: PeerJ. 2026 Feb 16;14:e20651. doi: 10.7717/peerj.20651 (PMC12919321; doi:10.7717/peerj.20651)
Supplement: Supplemental Information 1 — P hosphorous source, bacterial strain, and the interaction of both factors related to plant height, shoot diameter, and number of leaves in tomato (Solanum lycopersicum). [file peerj-14-20651-s001.docx]

**Supplementary information**

**Table 1.** Significance analysis of factors phosphorous source, bacterial strain, and the interaction of both factors related to plant height in tomato (*Solanum lycopersicum*).

| Factor | Plant height | | | |
| --- | --- | --- | --- | --- |
|  | 7 d | 14 d | 21 d | **28 d** |
| Phosphorous source | 0.0001* | 0.1895 | 0.8072 | **0.0001*** |
| Bacterial strain | 0.9612 | 0.9321 | 0.1603 | **0.0080*** |
| Phosphorous source* Bacterial strain | 0.0370* | 0.6466 | 0.1819 | **0.0054*** |

Significant differences p≤ 0.05 (*); Non-significant (ns).

**Table 2.** Significance analysis of factors phosphorous source, bacterial strain, and the interaction of both factors related to shoot diameter in tomato (*Solanum lycopersicum*).

| Factor | Shoot diameter | | | |
| --- | --- | --- | --- | --- |
|  | 7 d | 14 d | 21 d | 28 d |
| Phosphorous source | 0.01* | 0.20ns | 0.06ns | 0.003* |
| Bacterial strain | 0.28ns | 0.006* | 0.19ns | 0.07ns |
| Phosphorous source* Bacterial strain | 0.74ns | 0.22ns | 0.44ns | 0.20 ns |

Significant differences p≤ 0.05 (*); Non-significant (ns).

**Table 3.** Significance analysis of factors phosphorous source, bacterial strain, and the interaction of both factors related to number of leaves in tomato (*Solanum lycopersicum*).

| Factor | Number of leaves | | |
| --- | --- | --- | --- |
|  | 7 ddt | 14 ddt | 21 ddt |
| Phosphorous source | 0.36 | 0.17 | 0.004 |
| Bacterial strain | 0.46 | 0.06 | 0.0062 |
| Phosphorous source* Bacterial strain | 0.0008* | 0.80 | 0.0136 |

Significant differences p≤ 0.05 (*); Non-significant (ns).

**Table 4.** Significance analysis of factors phosphorous source, bacterial strain, and the interaction of both factors related to SPAD in tomato (*Solanum lycopersicum*).

| Factor | SPAD | | | |
| --- | --- | --- | --- | --- |
|  | 7 d | 14 ddt | 21 d | 28 d |
| Phosphorous source | 0.00* | 0.02* | <.0001* | <.0001* |
| Bacterial strain | 0.09ns | 0.50ns | 0.0041* | 0.01* |
| Phosphorous source* Bacterial strain | 0.45ns | 0.93ns | 0.0090* | 0.35ns |

Significant differences p≤ 0.05 (*); Non-significant (ns).

**Table 5.** Significance analysis of factors phosphorous source, bacterial strain, and the interaction of both factors related to Chlorophyll *a*, Chlorophyll *b*, and carotenoids in tomato (*Solanum lycopersicum*).

| Factor | Chlorophyll *a* | Chlorophyll *b* | Carotenoids |
| --- | --- | --- | --- |
| Phosphorous source | *0.0073 | *0.0421 | *0.0029 |
| Bacterial strain | 0.6041 | 0.4687 | *0.0160 |
| Phosphorous source* Bacterial strain | 0.2687 | 0.8407 | 0.1584 |

Significant differences p≤ 0.05 (*); Non-significant (ns).

**Table 6.** Significance analysis of factors phosphorous source, bacterial strain, and the interaction of both factors related to Leaf area, Aerial part freshweight, and Aerial part dry biomass in tomato (*Solanum lycopersicum*).

| Factor | Leaf area | Aerial part freshweight | Aerial part dry biomass |
| --- | --- | --- | --- |
| Phosphorous source | *0.0042 | 0.96 | 0.1680 |
| Bacterial strain | *0.0001 | *0.0001 | *0.0239 |
| Phosphorous source* Bacterial strain | *0.0004 | *0.001 | 0.0622 |

Significant differences p≤ 0.05 (*); Non-significant (ns).

**Table 7.** Significance analysis of factors phosphorous source, bacterial strain, and the interaction of both factors related to Root fresh biomass, Root dry biomass, Root volume, and Root length in tomato (*Solanum lycopersicum*).

| Factor |  | | | |
| --- | --- | --- | --- | --- |
|  | Root fresh biomass | Root dry biomass | Root volume | Root length |
| Phosphorous source | *0.0105 | *0.0001 | *0.0388 | *0.0035 |
| Bacterial strain | 0.2650 | *0.0116 | *0.0001 | *0.0089 |
| Phosphorous source* Bacterial strain | 0.3400 | 1.00 | *0.0021 | *0.0626 |

Significant differences p≤ 0.05 (*); Non-significant (ns).

**Table 8.** Significance analysis of factors phosphorous source, bacterial strain, and the interaction of both factors related to Concentration of nitrogen in aerial part, Concentration of phosphorous in aerial part, and Concentration of potassium in aerial part in tomato (*Solanum lycopersicum*).

| Factor | Concentration of nitrogen in aerial part | Concentration of phosphorous in aerial part | Concentration of potassium in aerial part |
| --- | --- | --- | --- |
| Phosphorous source | 0.0001* | 0.0001* | 0.5662ns |
| Bacterial strain | 0.9977ns | 0.22291ns | 0.4262ns |
| Phosphorous source* Bacterial strain | 0.0189* | 0.0079* | 0.3158ns |

Significant differences p≤ 0.05 (*); Non-significant (ns).
